# Controllable orbital angular momentum monopoles in chiral topological semimetals

**DOI:** 10.1038/s41567-024-02655-1

**Published:** 2024-09-30

**Authors:** Yun Yen, Jonas A. Krieger, Mengyu Yao, Iñigo Robredo, Kaustuv Manna, Qun Yang, Emily C. McFarlane, Chandra Shekhar, Horst Borrmann, Samuel Stolz, Roland Widmer, Oliver Gröning, Vladimir N. Strocov, Stuart S. P. Parkin, Claudia Felser, Maia G. Vergniory, Michael Schüler, Niels B. M. Schröter

**Affiliations:** 1 Laboratory for Materials Simulations, Paul Scherrer Institute, Villigen, Switzerland; 2 École Polytechnique Fédérale de Lausanne (EPFL), Lausanne, Switzerland; 3 Max Planck Institute of Microstructure Physics, Halle, Germany; 4 Max Planck Institute for Chemical Physics of Solids, Dresden, Germany; 5 Donostia International Physics Center, Donostia - San Sebastian, Spain; 6 Indian Institute of Technology-Delhi, New Delhi, India; 7 nanotech@surfaces Laboratory, Empa, Swiss Federal Laboratories for Materials Science and Technology, Dübendorf, Switzerland; 8 Swiss Light Source, Photon Science Division, Paul Scherrer Institute, Villigen, Switzerland; 9 Département de physique et Institut quantique, Université de Sherbrooke, Sherbrooke, Québec Canada; 10 Department of Physics, University of Fribourg, Fribourg, Switzerland; 11 Present Address: Laboratory for Muon Spin Spectroscopy, Paul Scherrer Institute, Villigen, Switzerland

**Keywords:** Topological matter, Spintronics, Electronic and spintronic devices, Electronic properties and materials, Surfaces, interfaces and thin films

## Abstract

The emerging field of orbitronics aims to generate and control orbital angular momentum for information processing. Chiral crystals are promising orbitronic materials because they have been predicted to host monopole-like orbital textures, where the orbital angular momentum aligns isotropically with the electron’s crystal momentum. However, such monopoles have not yet been directly observed in chiral crystals. Here, we use circular dichroism in angle-resolved photoelectron spectroscopy to image orbital angular momentum monopoles in the chiral topological semimetals PtGa and PdGa. The spectra show a robust polar texture that rotates around the monopole as a function of photon energy. This is a direct consequence of the underlying magnetic orbital texture and can be understood from the interference of local atomic contributions. Moreover, we also demonstrate that the polarity of the monopoles can be controlled through the structural handedness of the host crystal by imaging orbital angular moment monopoles and antimonopoles in the two enantiomers of PdGa, respectively. Our results highlight the potential of chiral crystals for orbitronic device applications, and our methodology could enable the discovery of even more complicated nodal orbital angular momentum textures that could be exploited for orbitronics.

## Abstract (web summary)

Chiral topological materials have been predicted to host orbital angular momentum monopoles, which can be useful for orbitronics applications. Now such monopoles have been imaged in chiral materials.

## Main

The motion of electrons in solids can be described by wave packets, which may have a self-rotation around their centre of mass. This self-rotation gives rise to an orbital magnetic moment that is proportional to the orbital angular momentum (OAM) of the Bloch states^1,2^. OAM currents have been proposed as a viable alternative to spin currents for manipulating and controlling magnetism in nanoscale memory devices by giving rise to large spin and orbital torques^3–8^. However, most recent investigations of orbital currents and the related orbital Hall effect have mainly focused on non-magnetic centrosymmetric materials where the OAM is suppressed in the equilibrium ground state and can be generated only with the application of an electrical field or at surfaces^9–11^. Magnetic materials, which have an intrinsic orbital moment, have also been shown to exhibit the orbital Hall effect^12,13^. The common key feature for a sizeable orbital Hall effect is a non-trivial momentum dependence of magnetic orbitals (known as orbital texture^14^), which can enable orbital currents even in centrosymmetric crystals^15,16^. In non-magnetic helical molecules and non-magnetic structurally chiral crystals, the Bloch bands have such an OAM texture in the ground state, which leads to orbital polarization when an electric field is applied. This can be exploited for launching and injecting large orbital currents and creating orbital polarization in orbitronic devices.

In helical molecules, this orbital polarization has recently been attributed to the observation of the chiral-induced spin selectivity effect^17^ due to the conversion of OAM to spin in contacts with strong spin–orbit coupling^18^. However, in contrast to chiral molecules, much less is known experimentally about the OAM textures in chiral crystals.

In chiral cubic crystals, crystalline symmetries can stabilize twofold and multifold degenerate band crossings at time-reversal invariant momenta of the Brillouin zone, which are known as Kramers–Weyl and multifold fermions^19,20^. These crossing points are expected to be strong sources and sinks of OAM, which results in large orbital Hall effects and current-induced orbital magnetization with a susceptibility that is predicted to be an order of magnitude larger than in corresponding Rashba systems^21,22^. Moreover, due to the high symmetry of cubic crystals and the absence of mirror symmetries in chiral systems, the direction of the OAM is expected to be locked isotropically parallel or antiparallel along the direction of the crystal momentum of the Bloch states in the vicinity of a linear band crossing point. As a result, the induced orbital polarization and magnetization depend only on the direction of the injected charge current and not on the crystallographic direction along which the charge current is flowing. Such an isotropic longitudinal magnetoelectric effect could be used for device applications, for instance, for switching magnetic domains with perpendicular magnetic anisotropy^21^. Rotational disorder of crystal grains is common in thin-film devices, and it could quench an anisotropic response. Thus, OAM monopoles with isotropic parallel OAM locking could be particularly valuable for device applications. As OAM is a pseudovector, an OAM monopole will be transformed into an antimonopole and vice versa upon reversing the structural chirality of the host crystal. Demonstrating this transformation law could highlight structural chirality as a design parameter for orbitronic applications because the polarity of the OAM monopole determines the directionality of transport responses. More generally, OAM monopoles are a manifestation of the quantum geometry of the electrons in periodic solids, which influences Fermi-liquid transport^23,24^ and opto-electronic properties^25–28^.

Multifold fermions have recently been observed with angle-resolved photoelectron spectroscopy (ARPES) in cubic chiral crystals with the B20 crystal structure^29–32^. However, these experiments investigated only the dispersion relationship of these multifold fermions and not their OAM texture. Therefore, so far, direct experimental evidence for the existence of OAM monopoles near Kramers–Weyl or multifold fermions in chiral crystals remains elusive. Moreover, it has not yet been demonstrated that the polarity of such OAM monopoles can be controlled through the structural chirality of the host crystal. Note that achiral Weyl semimetals, such as TaAs, can also host OAM monopoles^33^. However, due to the mirror symmetries present in these compounds, monopoles with opposite polarities are pinned to the same energies, which can lead to a cancellation of the effective orbital polarization, making them less valuable for orbitronics. In contrast, in chiral topological semimetals, monopoles with opposite polarity are generically separated in energy^31^, which has been predicted to result in large orbital polarizations^21,22^. Moreover, because band degeneracies cannot be pinned to high-symmetry points in achiral Weyl semimetals, they cannot exhibit isotropic OAM momentum locking, as for chiral topological semimetals.

Probing the OAM of Bloch electrons, with its full momentum dependence, to identify the presence of OAM monopoles still remains a grand challenge. As spin and orbital moments in solids influence the circular dichroism observed in many spectroscopic probes, such as X-ray circular dichroism, it seems natural to suspect that circular dichroism in ARPES (CD-ARPES), which is sensitive to time-reversal symmetry breaking^34,35^, might also be used to map out the momentum distribution of the OAM of Bloch states. Indeed, for the surface states of bulk Au (ref. ^9^) and Bi_2_Se_3_ (ref. ^36,37^) as well as for two-dimensional materials^38,39^, a qualitative agreement between CD-ARPES and the OAM (projected onto the incidence direction of light) has been observed. Although this apparent direct link from circular dichroism to the OAM texture, which is in turn closely related to the Berry curvature^2^, is the working hypothesis of recent experiments^33,40,41^, its general validity for bulk materials is far from clear. Details of the experimental geometry and complicated final states^42,43^ obscure the link. For instance, the reversal of circular dichroism in Bi_2_Te_3_ at different photon energies has been attributed to final-state effects^44^. Even for simple materials where the bands are dominated by the *d* orbital characteristics, the circular dichroism is not necessarily indicative of OAM^45,46^. For materials with several atoms per unit cell, (photon-energy-dependent) interference further complicates the signal^47,48^. Hence, to understand complex quantum materials, such as chiral topological semimetals hosting OAM monopoles^49^, a microscopic picture beyond the standard paradigm is required.

In this paper, we fill this gap for the chiral topological metals PdGa and PtGa with a blend of CD-ARPES experiments and photoemission simulations. Our calculations are based on a unique compromise between accurately reproducing the measured signal and the transparency of our model, which enabled us to interpret the complicated photon-energy dependence of our CD-ARPES spectra, thus revealing direct evidence for the presence of OAM monopoles in these chiral crystals.

## Results

**Fig. 1:**
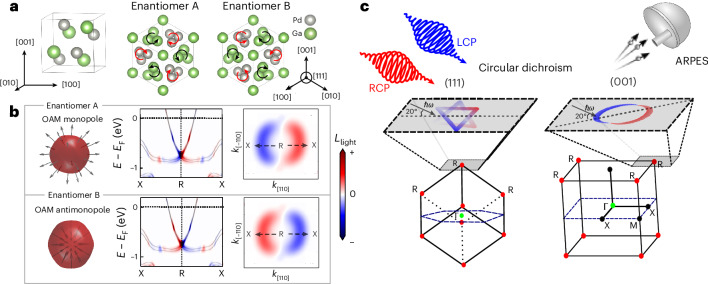
OAM monopoles in chiral topological semimetals PdGa/PtGa. **a**, Sketch of the chiral crystal structure of PdGa (B20 structure), highlighting the helical winding of the atoms along the [111] crystal direction. **b**, Calculated global OAM monopole texture around the double spin-1 multifold band degeneracy at the R point (left) in the corner of the Brillouin zone. The arrows on the sphere indicate the OAM direction on the constant energy contour at 30 meV above the crossing. Parallel OAM momentum locking projected onto the light direction (*L*_light_), along the X–R–X direction (middle, with binding energy *E* shifted by Fermi energy *E*_F_) and on the iso-energy surface in the X–R–M plane ~0.15 eV above the node (right, with momentum axis *k*_[110]_ along the [110] direction and *k*_[−110]_ along the [−110] direction). **c**, Illustration of the experimental geometry of the CD-ARPES experiment. Varying the crystal orientation allowed us to probe the three-dimensional OAM texture along different directions around the R point. LCP, left-handed circular polarization; RCP, right-handed circular polarization.

PdGa and PtGa crystallize in the structurally chiral B20 crystal structure (space group 198, 1a). The combination of crystalline symmetries and time-reversal symmetry protect a double spin-1 multifold fermion band crossing that is pinned to the R point in the corner of the cubic Brillouin zone^19,50,51^. For momenta close to the node, the Fermi surface is approximately spherical with isotropic parallel locking between the OAM and the crystal momentum, which can be observed in ab initio calculations of the global OAM of the Weyl bands in Fig. 1b,c. Similar polar features can be found in the local atomic OAM around all the atoms, which we discuss in the following sections (see Supplementary Information Note 6 for the conventions of OAM). As the two enantiomers are structurally related to each other through their mirror symmetry, the corresponding global OAM monopoles show opposite polar textures.

### Signature of orbital texture in CD-ARPES

To probe this polar OAM texture in PdGa and its related sister compound PtGa, we measured CD-ARPES Fermi surfaces on the (001) and (111) crystal planes (the corresponding measurement geometries are sketched in Fig. 1d). Following the traditional argument that CD-ARPES measures a projection of the OAM along the direction of the incident light^33,52,53^, one would expect that the Fermi-surface maps near the R point should display a polar CD-ARPES texture, that is a sign change for positive and negative momenta with respect to the R point, irrespective of the orientation of the crystal structure relative to the light direction or the photon energy used.

**Fig. 2:**
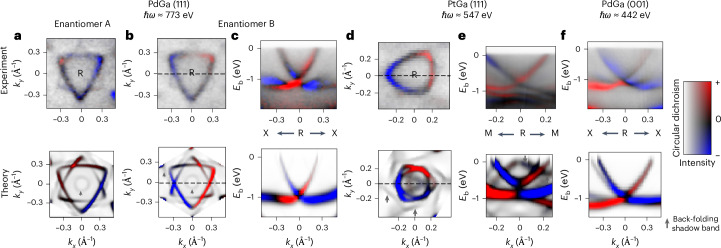
CD-ARPES near the R point in PdGa and PtGa. **a**–**f**, The projection of the photon momentum onto the sample surface is aligned with the *k*_*x*_ direction. **a**,**b**, Dichroic Fermi-surface data of PdGa(111) for enantiomer A (**a**) and enantiomer B (**b**). **c**, Binding-energy (*E*_b_)-dependent dichroic spectrum along the dashed line in **b**. **d**,**e**, Data for PtGa(111): *k*_*y*_ (**d**) and *E*_b_ (**e**). **f**, *E*_b_ for PdGa(001). The *k*_*x*_ direction in **e** (**b**,**f**) is aligned along M–R–M (X–R–X). Upper panels show experimental results. Lower panels show the corresponding calculated CD-ARPES spectra under the same conditions as in the experiments. The two-dimensional colour maps encode both the photoemission intensity and the circular dichroism. The [111] supercell calculation includes ‘shadow bands’, which are artefacts due to the finite size of the slab geometry. These artefacts are indicated with grey arrows.

Figure 2 presents the CD-ARPES measurements for the (111) surface of PtGa and PdGa (Fig. 2a–e) and for the (001) surface of PdGa (Fig. 2f). The incoming light momentum has a 20° grazing incidence angle with the *k*_*x*_ axis and is orthogonal to the *k*_*y*_ axis. All measured spectra are accompanied by simulated CD-ARPES spectra (lower panels, Fig. 2) based on the Wannier approach to ARPES^54–56^ (Methods). Although the complications of the final states are captured only approximately, the Wannier-ARPES method has been shown to qualitatively reproduce linear and circular dichroism for a range of systems^39,54,55^. The photon energies were carefully chosen such that the probed out-of-plane momentum corresponds to the plane containing the R point in the three-dimensional cubic Brillouin zone.

As Fig. 2 demonstrates, a polar texture of circular dichroism near the multifold fermion band crossing at the R point is ubiquitous throughout the different materials and orientations. In other words, there is a characteristic sign change of the CD-ARPES spectrum for positive and negative *k*_*x*_ momenta with respect to the R point. This observation is consistent with the conventional view that CD-ARPES measures the projection of the OAM monopole along the photon momentum. Moreover, this sign change is reversed for the Fermi surfaces of the two enantiomers (Fig. 2a,b), which is consistent with the expectation that the polarity of the OAM pole can be controlled with the handedness of the host crystal (see Supplementary Information Note 4 for a symmetry analysis of circular dichroism).

### Photon-energy dependence of the circular dichroism

**Fig. 3:**
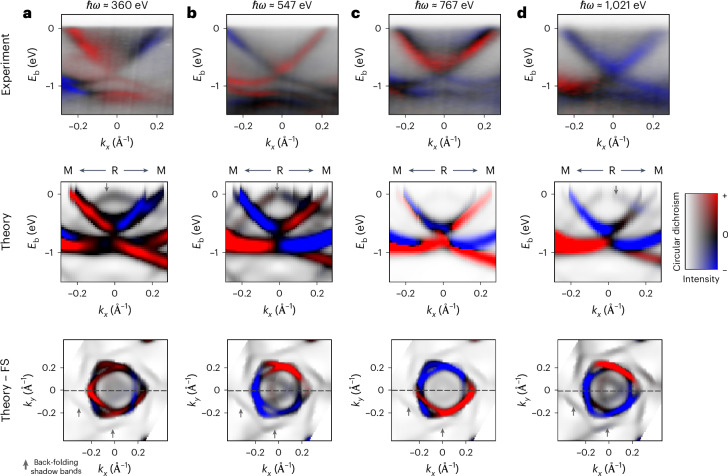
Photon-energy evolution of CD-ARPES spectra for PtGa (111). **a**–**d**, Energy versus momentum along the M–R–M direction for different photon energies: 360 eV (**a**), 547 eV (**b**), 767 eV (**c**) and 1,021 eV (**d**). Top row, Resolved by CD-ARPES. Middle row, Corresponding calculated spectra. Bottom row, Calculated CD-ARPES Fermi surfaces (FS). The dashed lines in the bottom row correspond to the line cuts in the middle row. The back-folded shadow-band artefacts have a visible intensity due to the finite slab size.

The simulated CD-ARPES spectra, which are in excellent qualitative agreement with the experiments, further support the robustness of the polar character. However, the simple picture of equating the sign change in the CD-ARPES spectra with a polar OAM texture is challenged when inspecting the photon-energy dependence (top row, Fig. 3). Taking PtGa(111) as a test case, there is a profound evolution of the CD-ARPES dispersions along the X–R–X direction when the photon energy is increased from *ℏω* = 360 eV (top row, Fig. 3a) towards higher soft-X-ray energies (top row, Fig. 3d). Strikingly, besides an overall sign inversion of the circular dichroism when going from *ℏω* = 360 to 547 eV, at the even higher photon energies of *ℏω* = 767 eV and *ℏω* = 1,021 eV the sign change of the CD-ARPES signal between positive and negative momenta with respect to the R point along the *k*_*x*_ direction seems to vanish completely. This unexpected photon-energy dependence shows that the CD-ARPES signal is not directly proportional to the total OAM associated with the Bloch wavefunction and raises the question whether there is any deeper connection between the predicted OAM monopole and the polar CD-ARPES spectrum observed at lower photon energies. We will see in the following that this is, indeed, the case and that the observed loss of sign change in the CD-ARPES signal along the *k*_*x*_ direction at higher photon energies is caused by photon-energy-dependent interference of local polar OAM textures from different atoms in the unit cell during the photoemission process.

Our simulations (middle row, Fig. 3) capture the essence of the evolution of the CD-ARPES spectra with the photon energy (albeit the deviations are larger at high photon energies). In particular, the overall sign reversal upon increasing the photon energy from *ℏω* = 360 to 565 eV (middle row, Fig. 3a,b) is clearly visible, and the loss of the sign change along the *k*_*x*_ axis at *ℏω* = 1,021 eV is also reproduced. Upon closer inspection of the simulated CD-ARPES Fermi surfaces (bottom row, Fig. 3), the effect of increasing the photon energy manifests as a rotation of the polar circular dichroism texture over the Fermi surface. That is, the CD-ARPES still changes sign for positive and negative momenta with respect to R but not symmetrically around the *k*_*x*_ = 0 plane. At higher photon energy, the *k*_*y*_ = 0 plane does not cut through the polar texture, resulting in the apparent loss of the characteristic sign change of the circular dichroism for positive and negative momenta in the experimental spectra (top row, Fig. 3c,d).

### Microscopic analysis of orbital texture and circular dichroism

The simulated CD-ARPES Fermi surfaces universally retain a polar structure as the key feature, and this can be linked to the predicted polar OAM texture around the multifold fermion at R. To understand this link, we analyse our simulations in detail. Starting from Fermi’s golden rule, the photoemission intensity with left-handed (+) and right-handed (−) circularly polarized light can be described as

1 $${I}^{\,(\pm )}({{\bf{k}}}_{\parallel },E\,)=\sum _{\alpha }\sum _{{k}_{\perp }}| {M}_{\alpha }^{\,(\pm )}({{\bf{k}}}_{\parallel },E\,){| }^{2}g({\varepsilon }_{\alpha }({\bf{k}})+\hslash \omega -E\,),$$

where ${M}_{\alpha }^{\,(\pm )}({{\bf{k}}}_{\parallel },E\,)$ denotes the matrix element for bloch band *α*, *g*(*ω*) denotes a broadened Dirac delta function containing the band energies (*ε*_*α*_(**k**)), the photon energy *ℏω*, the in-plane final-state momentum **k**_∥_ and the final-state energy *E*. The out-of-plane momentum *k*_⊥_ in equation (1) is determined from *E* and **k**_∥_ through

2 $$\frac{{{\bf{k}}}_{\parallel }^{2}}{2}+\frac{{k}_{\perp }^{2}}{2}=E-\varPhi,$$

where *Φ* denotes the work function.

We can express the key ingredient for computing CD-ARPES (the photoemission matrix element with respect to left-handed and right-handed circularly polarized photons, ${M}_{\alpha }^{\,(+)}({\bf{k}},E)$ and ${M}_{\alpha }^{\,(-)}({\bf{k}},E\,)$) in terms of the underlying orbitals. Constructing an effective Wannier Hamiltonian from first-principles density-functional theory (DFT) calculations provides us with the optimal representation of Bloch wavefunctions in terms of these orbitals (Methods). For PdGa, the Wannier model includes *d* orbitals localized on Pd sites and *p* orbitals at the Ga sites. Ignoring effects related to the finite escape depth of the photoelectrons^57^ (which mostly lead to further broadening), the circular dichroism can be expressed in terms of these orbitals in the bulk unit cell as

3 $$\begin{aligned}{\rm{CD}}({{\bf{k}}}_{\parallel },E\,)&\propto | {M}_{\alpha }^{\,(+)}({\bf{k}},E\,){| }^{2}-| {M}_{\alpha }^{\,(-)}({\bf{k}},E\,){| }^{2}\\ &\approx \sum\limits_{j{j}^{{\prime} }}{C}_{j\alpha }^{* }({\bf{k}}){T}_{j{j}^{{\prime} }}({\bf{k}}){C}_{{j}^{{\prime} }\alpha }({\bf{k}}).\end{aligned}$$

Here, *C*_*jα*_(**k**) describes the projection of Bloch state $\left\vert {\psi }_{{\bf{k}}\alpha }\right\rangle$ onto the orbital *j* and the tensor ${T}_{j{j}^{{\prime} }}({\bf{k}})$ incorporates the details of photoemission matrix elements with respect to all orbitals (C^*^_jα_ denotes the corresponding complex conjugate). The expansion in terms of the orbitals (equation (3)) allows us to dissect the various contributions to the circular dichroism. In particular, we can distinguish orbital-resolved, intra-atomic and interference contributions.

**Fig. 4:**
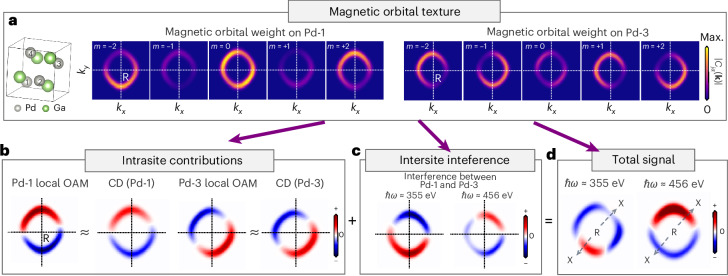
Orbital-resolved analysis of the circular dichroism. **a**, Texture of magnetic Pd *d* orbitals, at the two representative atomic sites Pd-1 and Pd-3, for *k*_*z*_ = 0 close to the R point. The quantization axis is identical to the incident light. **b**, Intrasite contributions from the Pd *d* orbitals, showing the qualitative equivalence of local OAM and the intra-atomic contributions to the circular dichroism (CD). **c**, Intersite interference contribution, here exemplified for the interference channel between the Pd-1 and Pd-3 atoms. **d**, Total bulk signal, obtained by summing all intrasite and interference contributions. Max., maximum.

Focusing on the region close to the multifold fermion (we chose 0.2 eV above the node), we found that mostly the Pd *d* orbitals contribute to the ARPES intensity. Each of the four Pd atoms carries momentum-dependent local OAM dictated by the chiral orbital texture of the magnetic quantum number *m* = −2, …, 2 with respect to the incidence direction, as shown in Fig. 4a (see Supplementary Information Note 6 for a definition of the local OAM). We first simulated the scenario where only a single selected Pd site contributed to the CD-ARPES signal. This was done by replacing *j* → *m* in equation (3) for the specific Pd atom and omitting all other terms, which we call local CD-ARPES. In this case, the local OAM is directly reflected in the (local) circular dichroism (Fig. 4b). When we then included all four Pd atoms but excluded interatomic interference effects, the total CD-ARPES signal was given by the sum of the dichroism originating from each of the Pd atoms, independent of the photon energy. The local OAM of each of Pd atoms remained polar, as for the global OAM, but exhibited a slightly different texture (Fig. 4b and Supplementary Information Note 6).

Finally, including interorbital interference terms, which have their specific polar texture, gave rise to a complex photon-energy-dependent pattern (for an example of two-atom interference, see Fig. 4c). The dependence on the photon energy can be understood by geometric arguments: the interference terms include a factor ${T}_{j{j}^{{\prime} }}({\bf{k}})\propto\operatorname{e}^{-\mathrm{i}{\bf{k}}\cdot ({{\bf{r}}}_{j}-{{\bf{r}}}_{{j}^{{\prime} }})}$ with **r**_*j*_ denoting the position of the Pd atoms in the unit cell. As the Pd atoms are at different vertical positions ${z}_{j}={[{{\bf{r}}}_{j}]}_{z}$ in the unit cell and out-of-plane photoelectron momentum *k*_⊥_ is photon-energy dependent (equation (2)), the interference terms attain a dependence on the photon energy.

From the measured circular dichroism, these complex contributions seem to impede the direct extraction of the global OAM, which contains the local OAM and the delocalized OAM of the Bloch states. Indeed, there is no simple specific rule, such as projecting the OAM along the incoming light direction, that governs the orientation of the resulting CD-ARPES iso-energy surfaces. However, the key ingredient determining both the global and local OAM monopoles—the asymmetric momentum-space texture of magnetic *d* orbitals (Fig. 4a)—manifests itself in all contributions to the simulated CD-ARPES pattern. Because every single contribution exhibits a polar texture, the resulting total CD-ARPES texture across the Fermi surface formed by interference will (except for an accidental cancellation at selected photon energies) generically show a polar texture as well. This means that there exists a line cut through *k* space that passes through the position of the OAM pole where the CD-ARPES signal changes sign for positive and negative momenta with respect to the OAM pole position. The direction of this line cut is not necessarily equivalent to the direction of the incoming light but can rotate around the position of the OAM pole depending on the photon energy. Hence, the polar structure of the circular dichroism that we observed for lower photon energies around the R point in Fig. 2 across different chiral crystals is a universal and relatively robust hallmark of the presence of OAM monopoles in our samples. This fingerprint is, in principle, universal and could be used to detect OAM monopoles in other samples as well.

Another important observation is that the signal at the same *k* point for energies above and below the multifold fermion at R in the global OAM (Fig. 1b) texture does not change sign, whereas the CD-ARPES spectra do sometimes have opposite signs. This difference in signs is not unexpected, because the CD-ARPES signal is not directly proportional to the OAM signal. The reason for the different signs above and below the node lies in the difference between the global OAM (defined by the modern theory of polarization) and the local OAM localized at the atomic sites. The global OAM includes the local contributions but also the delocalized OAM of the Bloch states^2^. Both contributions can have opposite signs, which is exactly what happens below the crossing point. Although the local contributions at the Pd or Pt sites exhibit a sign flip, which is consistent with the CD-ARPES signal, the global OAM does not have opposite signs for the bands below and above the node. However, the polar momentum-space texture over each iso-energy surface of the CD-ARPES signal is still evidence for the presence of a global OAM monopole at the R point, because both the global and the local OAM, as probed by CD-ARPES, share the same symmetry properties in momentum space.

To further underpin the key role of the chirality of the crystal structure for the formation of OAM monopoles, we performed the CD-ARPES simulations for the hypothetical closest crystal structure of PdGa with inversion symmetry in the bulk. As a result, the orbitals no longer carry a magnetic moment, and the weights of the ±*m* orbitals become equal. The band structure is still qualitatively similar, which allows a direct comparison. Although the simulated circular dichroism does not vanish due to the broken inversion symmetry at the surface, the polar texture is completely lost (Supplementary Information Note 5).

## Discussion

Identifying the OAM monopole is not trivial. From the theoretical side, DFT is not completely reliable at correctly predicting the electronic structure and orbital texture in some topological materials^58^. On the other hand, although the OAM is clearly manifest in CD-ARPES experiments, extracting the precise texture of the OAM—even on a qualitative level—is difficult. In particular, orbital interference and geometric interference, which are always present in complex quantum materials, break the direct correspondence between the circular dichroism and the OAM. For some materials, symmetry arguments can help identify characteristic sign changes in momentum space^33,40^, albeit only close to high-symmetry points. In general and especially for chiral materials as studied here, the symmetries cannot be exploited and the OAM texture is obscured, making it more challenging to directly detect the presence of OAM monopoles from CD-ARPES spectra.

Our work overcomes these challenges by making comprehensive measurements while we vary the crystal orientation and the photon energy. The results provide a detailed fingerprint of the underlying orbital texture. In particular, the monopole texture of the OAM around the R point is driven by a polar orbital texture, which manifests in a photon-energy-dependent CD-ARPES signal with a ubiquitous polar structure. Through the robustness of the polar circular dichroism, we have identified an unambiguous experimental signature of OAM monopoles in chiral materials. Moreover, the very good agreement of our CD-ARPES simulations with the experimental results implies that the isotropic radial OAM texture derived from DFT that was used in the CD-ARPES simulations is, indeed, the correct OAM texture for the multifold fermions in PtGa and PdGa. Moreover, our work also suggests that the large family of isostructural materials in the B20 crystal structure class, which feature similar electronic structures, could also host OAM monopoles.

This achievement raises the question whether other OAM textures, such as sources and sinks encountered at nodal lines^59,60^ or anti-crossings of Bloch bands that appear in many other quantum materials, can also be identified from CD-ARPES measurements. Based on our current findings, we expect that in the vicinity of such special points, there should also exist a characteristic texture of magnetic orbitals that should lead to a characteristic local circular dichroism. As supported by our analysis of the CD-ARPES signal in terms of the interference channels, sign changes in the circular dichroism that are robust against varying the photon energy or the crystal orientation are expected to provide a unique fingerprint of such OAM textures. Thus, our work utilizing CD-ARPES paves the path to directly measuring key features of momentum-resolved OAM textures that could lead to interesting magneto-transport and optical responses in a variety of quantum materials.

Finally, we also have shown that reversing the handedness of the crystal structure leads to a reversal of the polarity of the OAM monopoles in a crystal, which in turn is expected to change the sign of the (inverse) orbital Edelstein effect. This observation establishes structural chirality as a control parameter of OAM in charge-conversion processes. We anticipate that changing the handedness of chiral electrodes may, therefore, help to elucidate the origin of recently observed orbitronic effects, such as the long-range ballistic orbital currents^61^.

## Methods

### Crystal growth

PdGa and PtGa single crystals were grown from the melt with the self-flux technique described in refs. ^32,62^. After aligning the crystals at room temperature with a white-beam backscattering Laue X-ray set-up, the surfaces along different high-symmetry directions were polished.

### ARPES measurements

For the ARPES experiments, the crystal surfaces were cleaned in situ with multiple sputtering (using Ar^+^ at 1 keV for 20 min under 1 × 10^−5^ mbar) and annealing (>870 K for ≳20 min), as described in refs. ^32,63^. Soft X-ray ARPES experiments were performed at the SX-ARPES end station^64^ of the ADRESS beamline^65^ at the Swiss Light Source, Switzerland, using a PHOIBOS-150 (SPECS) analyser with the sample held at around 20 K under a pressure lower than 2 × 10^−10^ mbar. The angular resolution was about 0.1°, and the combined analyser and beamline energy resolution ranged from 60 to 180 meV for photon energies between 360 eV and 1.021 keV. Measurements were acquired with left-handed and right-handed circular polarization and normalized by the total intensity (${I}_{{\rm{tot}}}^{\pm }$) within the full measured range of the displayed two-dimensional spectra, before calculating the dichroism and total intensity:

4 $$\mathrm{CD}=\frac{{I}^{+}}{{I}_{{\rm{tot}}}^{+}}-\frac{{I}^{-}}{{I}_{{\rm{tot}}}^{-}},\quad {I}_{{\rm{tot}}}=\frac{1}{2}\left(\frac{{I}^{+}}{{I}_{{\rm{tot}}}^{+}}+\frac{{I}^{-}}{{I}_{{\rm{tot}}}^{-}}\right).$$

In addition, for the spectral cuts (Fig. 3a–d), *I*_tot_ was subsequently corrected for the analyser transmission (estimated by energy-integrating the spectra) and the angle-integrated background was subtracted. The intensity in all Fermi-surface iso-energy maps was integrated within ±100 meV of the Fermi level.

### DFT calculations and Wannier tight-binding Hamiltonian of PdGa/PtGa

The ground-state band structure of PdGa/PtGa was obtained from DFT calculations with the QUANTUM ESPRESSO code^66^. We chose the generalized gradient approximation of the Perdew–Burke–Ernzerhof functional^67^ for the exchange-correlation functional without spin–orbit coupling.

We constructed Wannier functions for the bulk structure of both compounds with the Wannier90 code^68^. We used the projective Wannier function approach without maximal localization to optimize the match of the Wannier functions with atom-like orbitals with well-defined angular quantum numbers. From the bulk Wannier Hamiltonian, we constructed a slab supercell of 15 layers for the (001) surface and 20 layers for the (111) surface. The slab Hamiltonian was then used in the Wannier-ARPES calculations.

### Wannier-ARPES simulations

To evaluate the photoemission intensity with equation (1), we need to compute the matrix elements ${M}_{\alpha }({{\bf{k}}}_{\parallel },E\,)=\langle {\chi }_{{{\bf{k}}}_{\parallel },E}| {\bf{e}}\cdot \hat{{\bf{r}}}| {\psi }_{{\bf{k}}\alpha }\rangle$ with respect to a given light polarization **e**, where $|\psi_{\vec{k}\alpha}\rangle$ is the Bloch state *α* and $|\chi_{\vec{k_{\parallel}}, E} \rangle$ is the photoemission final state with parallel crystal momentum $\vec{k_{\parallel}}$ and energy *E*. Here we represent the light–matter coupling in the dipole gauge. The dipole operator $\hat{{\bf{r}}}$ is evaluated using the modern theory of polarization^69^, which avoids any ill-definedness^56^. We further apply the atomic-centred approximation (ACA), which reduces the calculation of the photoemission matrix element to a summation over atomic transition amplitudes:

5 $${M}_{\alpha }({{\bf{k}}}_{\parallel },E\,)=\sum _{jl}{C}_{jl\alpha }({{\bf{k}}}_{\parallel })\operatorname{e}^{-\mathrm{i}{\bf{k}}\cdot {{\bf{r}}}_{jl}}\operatorname{e}^{{z}_{jl}/\lambda }{M}_{j}^{{\,\rm{(ACA)}}}({{\bf{k}}}_{\parallel },E\,)\,.$$

Here, *C*_*jlα*_(**k**_∥_) are the coefficients of Wannier orbital *j* in slab layer *l*, and **r**_*jl*_ denotes the position of the Wannier centre in the system (the *z* projection is given by *z*_*jl*_). The mean free path of the photoelectrons in the crystal *λ* determines the *k*_⊥_ broadening in the simulations. For clarity we focus on the case *λ* → *∞*.

The atomic transition amplitudes are defined as

6 $${M}_{j}^{{\,\rm{(ACA)}}}({{\bf{k}}}_{\parallel },E\,)=\int\,\mathrm{d}{\bf{r}}\,{\chi }_{{{\bf{k}}}_{\parallel },E}^{* }({\bf{r}}){\bf{e}}\cdot {\bf{r}}{\phi }_{j}({\bf{r}}),$$

where *ϕ*_*j*_(**r**) is the Wannier function of orbital *j*. It is convenient to expand both the orbitals and the final states in spherical harmonics with respect to the Wannier centre (which has been shifted to the origin in equation (6)). Approximating the Wannier orbitals as atom-like wavefunctions, ${\phi}_{j}({\bf{r}})={R}_{j}(r){Y}_{{{\ell}_{j}}{m}_{j}}({\Omega}_{\bf{r}})$, where ${Y}_{{{\ell}_{j}}{m}_{j}}({\Omega}_{\rm{r}})$ is the real spherical harmonic function, the atomic matrix elements are then given by

7 $${M}_{j}^{\,\rm{(ACA)}}({\bf{k}}_{\parallel},E\,)=4\uppi \sum_{\ell m}{(-i)}^{\ell}{Y}_{\ell m}({{\Omega}}_{\bf{k}}){C}_{{\ell m,}{\ell}_{j}{m}_{j}}({\bf{e}}){I}_{j,\ell}(E\,).$$

Here, the coefficients ${C}_{\ell m,{\ell }_{j}{m}_{j}}({\bf{e}})$ are defined in terms of the Clebsch–Gordan coefficients, which incorporate the dipole selection rules *ℓ* = *ℓ*_*j*_ ± 1 and *m* = *m*_*j*_ and *m*_*j*_ ± 1 depending on the light polarization **e**. The remaining unknown ingredients are radial integrals *I*_*j*,*ℓ*_(*E*). Their absolute value for different orbitals accounts for the relative transition strength, and their phase incorporates the phase shift of the partial waves from different orbitals and atoms.

We computed ${I}_{j,\ell = {\ell }_{j}\pm 1}(E)$ using the the Korringa–Kohn–Rostoker formalism^70^, which allows us to perform the expansion exactly in spherical harmonics around each atom for both the initial and the final states. From this calculation, we estimated $| {I}_{j,\ell = {\ell }_{j}\pm 1}(E)|$, which revealed that (1) the contribution from the Ga atoms can be neglected and (2) that the transition *d* → *f*, for example, ${I}_{j,\ell = {\ell }_{j}+1}(E)$ for Pd/Pt *d* orbitals (*ℓ*_*j* _= 2) dominates. For this reason, intra-atomic interference between the partial waves plays only a minor role.

For convenience, we parameterized the radial integrals:

8 $${I}_{j,\ell }(E\,)=\int_{0}^{\infty}\,\mathrm{d}r\,{r}^{3}{j}_{\ell }(kr){R}_{j}(r),$$

which is reminiscent of the expression within the plane-wave approximation. We used equation (8) to interpolate to the radial integrals over the required energy range by adjusting the radial wavefunctions *R*_*j*_(*r*). In practice, we represent *R*_*j*_(*r*) by Slater-type orbitals with principal quantum number *n* = 4 and optimize the effective charge *Z*_*j*_ such that the parametrization in equation (8) is consistent with the Korringa–Kohn–Rostoker results. The details are discussed in the Supplementary Information. All calculations were performed using the DYNAMICS-W90 code^71^.

For Fig. 4b–d, the circular dichroism was calculated with the simplified photoemission model in equation (3) using the bulk Wannier Hamiltonian. The simulation was performed at *k*_⊥_ = π/*a* with a map on different **k**_∥_, where *a* is the cubic lattice constant. The result qualitatively reproduces the measured circular dichroism. In particular, the cut along the X–R–X direction in Fig. 4d is in excellent agreement with the CD-ARPES map in Fig. 2c (lower row). To improve the quantitative agreement and obtain the results presented in Figs. 2 and 3, we included the presence of the surface, which we did by simulating ARPES for an entire slab. Although the details of the spectra change, the salient polar texture was only weakly affected, underlining the orbital picture outlined above.

## Online content

Any methods, additional references, Nature Portfolio reporting summaries, source data, extended data, supplementary information, acknowledgements, peer review information; details of author contributions and competing interests; and statements of data and code availability are available at 10.1038/s41567-024-02655-1.

## Supplementary information

Supplementary Information Supplementary Notes 1–7, Equations 1–20 and Figs. 1–5.

## Data Availability

The data used in this study is available on the Open Research Data Repository of the Max Planck Society (10.17617/3.PILCPQ).

## Code availability

The DFT calculations were performed with the QUANTUM ESPRESSO code^66^. The ARPES simulation was performed with our open source code DYNAMICS-W90, which is available from GitHub (https://github.com/michaelschueler/dynamics-w90)^71^. ARPES data were analysed with the MATools package, which is available from https://www.psi.ch/en/sls/adress/manuals, and custom scripts, which are available in the data repository at 10.17617/3.PILCPQ. Fig. 1a was plotted with VESTA^72^.
