# Supplementary material for: Controllable orbital angular momentum monopoles in chiral topological semimetals
Source: Nat Phys. 2024 Sep 30;20(12):1912–8. doi: 10.1038/s41567-024-02655-1 (PMC11631750; doi:10.1038/s41567-024-02655-1)
Supplement: Supplementary file 1 — Supplementary Notes 1–7, Equations 1–20 and Figs. 1–5. [file 41567_2024_2655_MOESM1_ESM.pdf]

nature physics

Supplementary information https://doi.org/10.1038/s41567-024-02655-1

Controllable orbital angular momentum monopoles in chiral topological semimetals

In the format provided by the authors and unedited

Supplementary Information: Controllable orbital angular momentum monopoles in chiral topological semimetals

Yun Yen,[1,2,*] Jonas A. Krieger,[3,*,†] Mengyu Yao,[4,*] Iñigo Robredo,[4,5] Kaustuv Manna,[6,7] Qun Yang,[6] Emily C. McFarlane,[3] Chandra Shekhar,[6] Horst Borrmann,[6] Samuel Stolz,[8] Roland Widmer,[8] Oliver Gröning,[8] Vladimir N. Strocov,[9] Stuart S.P. Parkin,[3] Claudia Felser,[6,‡] Maia G. Vergniory,[6,5] Michael Schüler,[1,10,§] and Niels B. M. Schröter[3,¶]

[1]*Laboratory for Materials Simulations, Paul Scherrer Institute, Villigen PSI, Switzerland*
[2]*École Polytechnique Fédérale de Lausanne (EPFL), Switzerland*
[3]*Max Planck Institut für Mikrostrukturphysik, Weinberg 2, 06120 Halle, Germany*
[4]*Max Planck Institute for Chemical Physics of Solids, Dresden, Germany*
[5]*Donostia International Physics Center, 20018 Donostia - San Sebastian, Spain*
[6]*Max Planck Institute for Chemical Physics of Solids, Dresden, Germany*
[7]*Indian Institute of Technology-Delhi, Hauz Khas, New Delhi 110 016, India*
[8]*nanotech@surfaces Laboratory, Empa, Swiss Federal Laboratories for Materials Science and Technology, 8600 Dübendorf, Switzerland*
[9]*Photon Science Division, Paul Scherrer Institute, 5232 Villigen PSI, Switzerland*
[10]*Department of Physics, University of Fribourg, Fribourg, Switzerland*

Contents

Supplementary Note 1. Out-of-plane dispersion 1

Supplementary Note 2. Matching the radial integral to KKR calculation 2

Supplementary Note 3. PdGa(001) photon energy dependent circular dichroism 3

Supplementary Note 4. Symmetry relation of circular dichroism in two enantiomers 3

Supplementary Note 5. Circular dichroism in inversion symmetric PdGa 4

Supplementary Note 6. Global and local Orbital Angular Momentum 4

Supplementary Note 7. Correspondence between local OAM and local CD 5

References 7

Supplementary Note 1. OUT-OF-PLANE DISPERSION

The out-of-plane momentum corresponding to a photoelectron with kinetic energy E_{kin} was calculated as:

$$k_z = \sqrt{\frac{2m_{\mathrm{e}}\left(E_{\mathrm{kin}} - V_{000}\right)}{\hbar^2} - k_{\parallel}^2} + p_{\gamma,z}, \qquad \text{(Supplementary Equation S1)}$$

where m_{e} is the electron mass, $k_{\parallel}$ the in-plane momentum, V_{000} the inner potential and $p_{\gamma,z}$ the component of the photon momentum ($h\nu/c$) perpendicular to the sample surface. In particular, we used the expressions specifically derived for the specific measurement geometry of ADRESS in Ref. [S1]. We have estimated the inner potential of

---

* These authors contributed equally to this work
† Current address: Laboratory for Muon Spin Spectroscopy, Paul Scherrer Institute, CH-5232 Villigen PSI, Switzerland
‡ Electronic address: claudia.felser@cpfs.mpg.de
§ Electronic address: michael.schueler@psi.ch
¶ Electronic address: niels.schroeter@mpi-halle.mpg.de

PtGa and PdGa to be $V_{000} \approx 12\,\mathrm{eV}$ by comparing the measured band structure with the expected periodicity of the Brillouin zone shown as solid lines in Fig. S1.

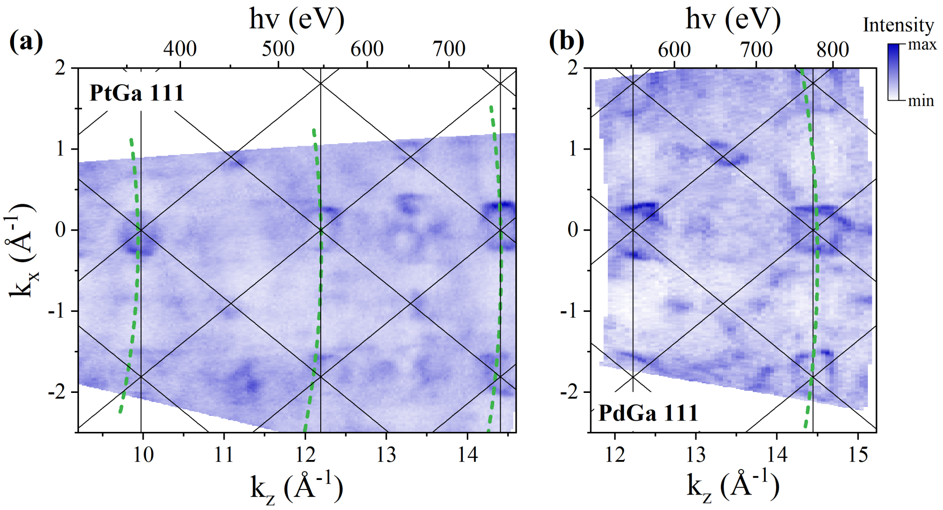

Supplementary Figure S1: **Out-of-plane dispersion on the 111 surfaces.** (a) Fermi surface map with the top axis showing the photon energy at $k_x = 0\,\text{Å}^{-1}$. (b) The same for PdGa 111. The expected Brillouin-zone boundaries are drawn as black lines. The green dashed lines show the constant photon energy cuts corresponding to the Fermi surfaces and spectra shown in the main paper.

Supplementary Note 2. MATCHING THE RADIAL INTEGRAL TO KKR CALCULATION

The photoelectron radial matrix elements were calculated with the full relativistic Munich SPRKKR code (Version 7.7) [S2–S4] for photon energies ($\hbar\omega$) in the range of 350 to 550 eV. As a basis, the electronic structure of PdGa including potential and electron wave functions were calculated using the exchange–correlation functional in the parameterization of Perdew *et al.* [S5]. Using the relativistic KKR Kohn-Sham radial wave functions, the radial matrix elements were computed in the relativistic $\vec{\alpha}\cdot\vec{A}$-form ($\vec{\alpha}$ and $\vec{A}$ are the Dirac matrix and the vector potential, respectively). In particular, the following transitions were considered for emission from Pd d-states: $p_{1/2} \leftarrow d_{3/2}$, $p_{3/2} \leftarrow d_{3/2}$, , $p_{3/2} \leftarrow d_{5/2}$, $f_{5/2} \leftarrow d_{5/2}$, and $f_{7/2} \leftarrow d_{5/2}$. In the investigated photon energy range, the larges matrix elements were obtained for $f_{5/2} \leftarrow d_{3/2}$ and $f_{7/2} \leftarrow d_{5/2}$ transitions. At $\hbar\omega = 440$ eV, These transitions exhibit the largest variation over the valence band. The remaining transitions are varying less but are clearly smaller.

With this input, we have build a model for the radial integrals $I_{j,\ell}(E)$ entering the Wannier-ARPES calculations (Eq. (7) in the main text). For computational convenience, we paramterize the radial integrals by a model inspired by the plane-wave approximation, which is fitted against the KKR results for $|I_{j,\ell}(E)|$. Operationally, we approximate the radial functions $R_j(r)$ defining the atom-like Wannier functions $\phi_j(\vec{r}) = R_j(r)Y_{\ell_j m_j}(\Omega_{\vec{r}})$ by Slater-type orbitals $R(\ell, n, Z; r)$, and write the radial integral as

$$I_{j,\ell} = \int_0^\infty dr\, r^3 j_\ell(pr) R^{\mathrm{sl}}(\ell_j, n_j, Z_j; r) \ . \qquad \text{(Supplementary Equation S2)}$$

Here, Z_j denotes the effective charge, while n_j is the principal quantum number fixed at $n_j = 4$ for the Pd/Pt 4d orbitals. $j_\ell(pr)$ is spherical Bessel function with angular momentum ℓ with final state momentum $p = \sqrt{2E}$. We compare $I_{j,\ell}(E)$ for different values for Z_j to the KKR results in Fig. S2 for the example of the Pd-4d orbital, where the only two channels are d $\rightarrow$ p and d $\rightarrow$ f are allowed by the dipole selection rule. The radial integral with $Z_j = 8.0$ has similar photon energy dependence as KKR. In Fig. S2**c**, the ratio between two channels are close to 4 for both KKR and $Z =_j 8.0$ calculation, indicating that the d $\rightarrow$ f channel is dominant in the photoemission process.

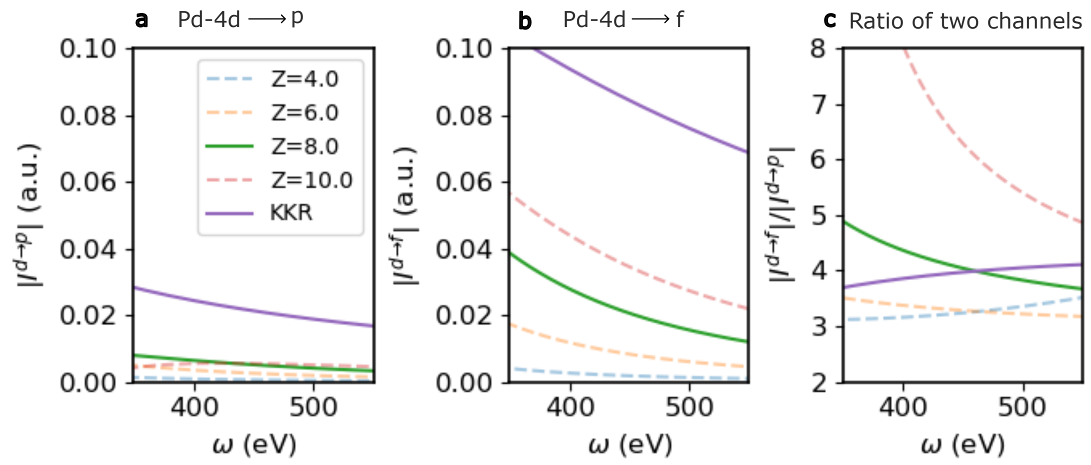

Supplementary Figure S2: **Radial integrals for KKR calculation and slater-type orbitals a** Absolute value of the radial intensity for transition from Pd-4d orbitals to p-type final states. **b** Absolute value of the radial intensity for transition from Pd-4d orbitals to f-type final states. **c** Ratio between two channels.

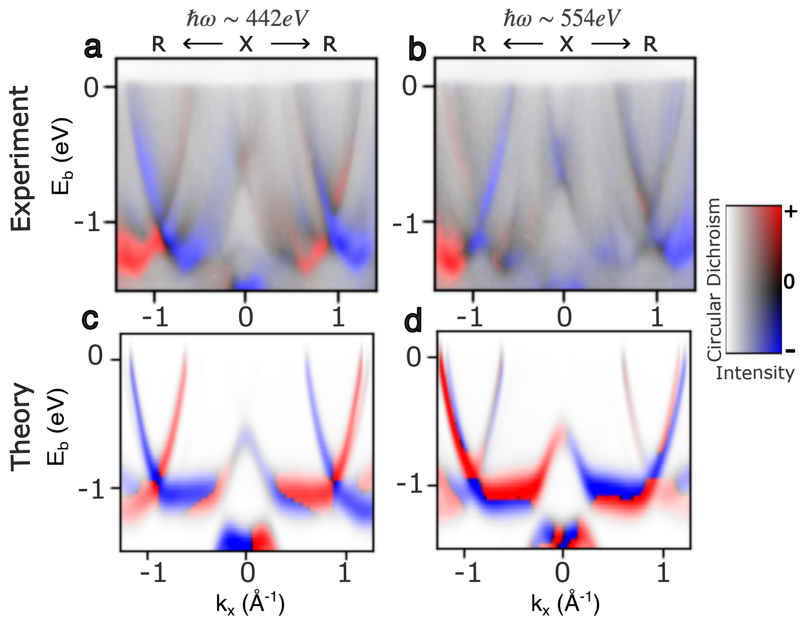

Supplementary Figure S3: **CD photon energy dependence in PdGa(001) a,b** Experimental and **c,d** simulated CD in PdGa(001) agree well with each other.

Supplementary Note 3. PdGa(001) PHOTON ENERGY DEPENDENT CIRCULAR DICHROISM

In Fig. S3, the photon energy dependence of CD in PdGa(001) also shows agreement between experiment and theory, just like in PtGa(111) (Fig. 3 in the main text). The data shows the CD sign change as a function of photon energy and can be understood as the ”rotation” of polar CD signal (Fig. 4 in the main text).

Supplementary Note 4. SYMMETRY RELATION OF CIRCULAR DICHROISM IN TWO ENANTIOMERS

Suppose H_A and H_B are Hamiltonian for enantiomers A and B. We set the mirror plane as the same as the scattering plane, which is the y-z plane in our coordinate system. As a result, mirror symmetry operator $\hat{M}_x$ relates

the two enantiomers. Therefore we have

$$\hat{M}_x{}^{\dagger} H_A(\mathbf{k}) \hat{M}_x = H_B(\hat{M}_x \mathbf{k}) \quad \text{(Supplementary Equation S3)}$$

$$\hat{M}_x \left|\psi_\alpha^A(\mathbf{k})\right\rangle = \left|\psi_\beta^B(\hat{M}_x \mathbf{k})\right\rangle \quad \text{(Supplementary Equation S4)}$$

, where α and β label Bloch bands for A/B.

On the other hand, the dipole matrix elements for A/B can be written as

$$\langle \chi^B | \hat{\mathbf{r}} | \psi_\beta^B(\hat{M}_x \mathbf{k}) \rangle \quad \text{(Supplementary Equation S5)}$$

$$= \langle \chi^A | \hat{M}_x^\dagger \hat{\mathbf{r}} \hat{M}_x | \psi_\alpha^A(\mathbf{k}) \rangle \quad \text{(Supplementary Equation S6)}$$

$$= \langle \chi^A | (-\hat{\mathbf{x}}, \hat{\mathbf{y}}, \hat{\mathbf{z}}) | \psi_\alpha^A(\mathbf{k}) \rangle \quad \text{(Supplementary Equation S7)}$$

, where $\left|\chi^{A(B)}\right\rangle$ denotes the photoelectron final state for enantiomer A(B) with the relation $\left|\chi^B\right\rangle = \hat{M}_x \left|\chi^A\right\rangle$. Now with our exerimental geometry (light is 20° away from crystal plane), the light-matter interacting matrix elements in dipole gauge are simply

$$D_B^+(\hat{M}_x \mathbf{k}) = \mathbf{e}^+ \cdot \langle \chi^B | \hat{\mathbf{r}} | \psi_\beta^B(\hat{M}_x \mathbf{k}) \rangle \quad \text{(Supplementary Equation S8)}$$

$$= +iD^{Bx}(\hat{M}_x \mathbf{k}) + sin(20^o) D^{By}(\hat{M}_x \mathbf{k}) + cos(20^o) D^{Bz}(\hat{M}_x \mathbf{k}) \quad \text{(Supplementary Equation S9)}$$

$$= -iD^{Ax}(\mathbf{k}) + sin(20^o) D^{Ay}(\mathbf{k}) + cos(20^o) D^{Az}(\mathbf{k}) = D_A^-(\mathbf{k}) \quad \text{(Supplementary Equation S10)}$$

$$D_B^-(\hat{M}_x \mathbf{k}) = \mathbf{e}^- \cdot \langle \chi^B | \hat{\mathbf{r}} | \psi_\beta^B(\hat{M}_x \mathbf{k}) \rangle \quad \text{(Supplementary Equation S11)}$$

$$= -iD^{Bx}(\hat{M}_x \mathbf{k}) + sin(20^o) D^{By}(\hat{M}_x \mathbf{k}) + cos(20^o) D^{Bz}(\hat{M}_x \mathbf{k}) \quad \text{(Supplementary Equation S12)}$$

$$= +iD^{Ax}(\mathbf{k}) + sin(20^o) D^{Ay}(\mathbf{k}) + cos(20^o) D^{Az}(\mathbf{k}) = D_A^+(\mathbf{k}) \quad \text{(Supplementary Equation S13)}$$

, where $\mathbf{e}^{+(-)}$ represents right(left) circularly polarization. $D^{A(B)\mu} = \langle \chi^{A(B)} | \hat{\mu} | \psi_{\alpha(\beta)}^{A(B)}(\mathbf{k}) \rangle$ is the matrix element for cartesian direction μ. Finally we find that

$$CD_A(\mathbf{k}) = -CD_B(M_x \mathbf{k}). \quad \text{(Supplementary Equation S14)}$$

In Fig. 2**a** of the main text, the dichroic Fermi surface of the enantiomer A is measured with light direction 60° differed from enantiomer B (Fig. 2**b**). The matching simulation in Fig. 2**g** and **h** uses the same enantiomer with corresponding light geometry. Next we transform CD in enantiomer A (Fig. 2**g**) using the symmetry relation (S14). The agreement between experiment and simulation confirms the symmetry analysis.

Supplementary Note 5. CIRCULAR DICHROISM IN INVERSION SYMMETRIC PdGa

The observed photon energy dependent polar CD is a direct manifestation of OAM monopoles and chiral orbital texture, as discussed in the main text. To support this, we manually restore inversion symmetry in PdGa as in Fig. S4**B** and simulated the corresponding CD. In the bulk band structure (Fig. S4**A**), the crossings at R points are no longer Weyl points or OAM monopoles. As a result, local CD (Fig. S4**C-D**), inter-site interference terms (Fig. S4**E-F**), and the total CD (Fig. S4**G-H**) lose the polar nature.

Supplementary Note 6. GLOBAL AND LOCAL ORBITAL ANGULAR MOMENTUM

Orbital angular momentum (OAM) for periodic solids is rigorously defined in terms of the modern theory of magnetization [S6]:

$$L_\alpha^\mu(\mathbf{k}) = -i\epsilon_{\mu\nu\gamma} \sum_{\alpha\neq\alpha'} (\varepsilon_{\alpha'}(\mathbf{k}) - \varepsilon_\alpha(\mathbf{k})) A_{\alpha\alpha'}^\nu(\mathbf{k}) A_{\alpha'\alpha}^\gamma(\mathbf{k}) \ . \quad \text{(Supplementary Equation S15)}$$

Here, α denotes the band index, μ stands for the cartesian directions, and $\epsilon_{\mu\nu\gamma}$ is the Levi-Civita tensor. Besides the band structure $\varepsilon_\alpha(\mathbf{k})$, the Berry connections $A_{\alpha\alpha'}^\nu(\mathbf{k}) = i\langle u_{\mathbf{k}\alpha} | \partial_\nu u_{\mathbf{k}\alpha'} \rangle$ are the central ingredients to Eq. (S16). The

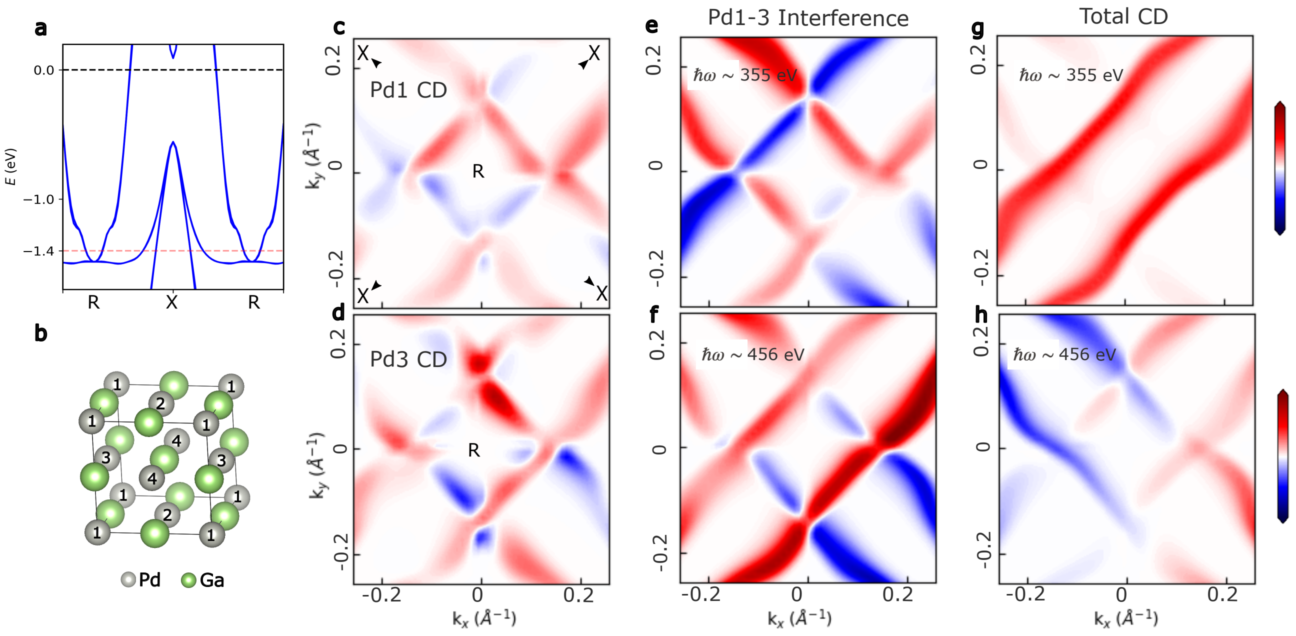

Supplementary Figure S4: **Inversion-symmetric PdGa simulation a** Band structure of **b** the inversion-symmetric PdGa. **c**,**d** Local CD from Pd1 and Pd3 atoms. **e**,**f** inter-site interferece between Pd1 and Pd3 at different photon energies. **G**,**H** Total CD signal with summation over every intra-site term and inter-site interference terms.

modern theory of OAM is closely tied to Berry curvature [S6]; indeed, if there are only two two bands, both quantities are proportional to each other [S7].

While the modern theory definition (S16) defines a *global* OAM that is proportional to the magnet moment of the crystal, ARPES experiments in the measurement geometry used in this work are sensitive to the *local* OAM at the different atomic sites. In terms of the Wannier Hamiltonian, the local OAM is defined as

$$L^{\mu}_{a,\alpha}(\mathbf{k}) = \sum_{m',m} C^{*}_{(am)\alpha}(\mathbf{k}) L^{\mu}_{mm'} C_{(am')\alpha}(\mathbf{k}) \ , \qquad \text{(Supplementary Equation S16)}$$

where m, m' run through magnetic orbitals localized at atomic site a. $L^{\mu}_{mm'}$ is the atomic OAM matrix elements, which depends on our choice of the quantization axis. Due to the broken translational invariance in the out-of-plane direction, the momentum derivative $\partial_z = \partial/\partial k_z$ reduces to the standard dipole operator in z-direction. As a consequence, the site-resolved circular dichroism corresponds to the local OAM at respective atomic site.

In Fig. 1**b**,**c** in the main text we show the global OAM from the modern theory (S15). The global OAM – and the Berry curvature – exhibit the hedgehog texture in the vicinity of the R-points. This hedgehog structure is also imprinted onto the local OAM at each atomic site in the unit cell, which is reflected in the polar structure of the local OAM in Fig.S5.

Supplementary Note 7. CORRESPONDENCE BETWEEN LOCAL OAM AND LOCAL CD

Fig.2 and Fig.3 in the main text demonstrate the remarkable agreement between the experiment and theory, which indicates the applicability of atomic centered approximation (ACA) in this work. In this section, we analyze how local intra-site CD computed with the ACA corresponds to the the local OAM, in the case of d orbital character bands. We rewrite the expression of circular dichroism in the case of infinite escape depth $\lambda \to \infty$ as

$$\begin{aligned} \mathrm{CD}(\mathbf{k},E) &\propto \left| \sum_j C_{j\alpha}(\mathbf{k}) e^{-i\mathbf{k}\cdot\mathbf{r}_j} M^{(+)}_j(\mathbf{k},E) \right|^2 - \left| \sum_j C_{j\alpha}(\mathbf{k}) e^{-i\mathbf{k}\cdot\mathbf{r}_j} M^{(-)}_j(\mathbf{k},E) \right|^2 \\ &= \sum_{jj'} C^{*}_{j\alpha}(\mathbf{k}) T_{jj'}(\mathbf{k},E) C_{j'\alpha}(\mathbf{k}) \ , \qquad \text{(Supplementary Equation S17)} \end{aligned}$$

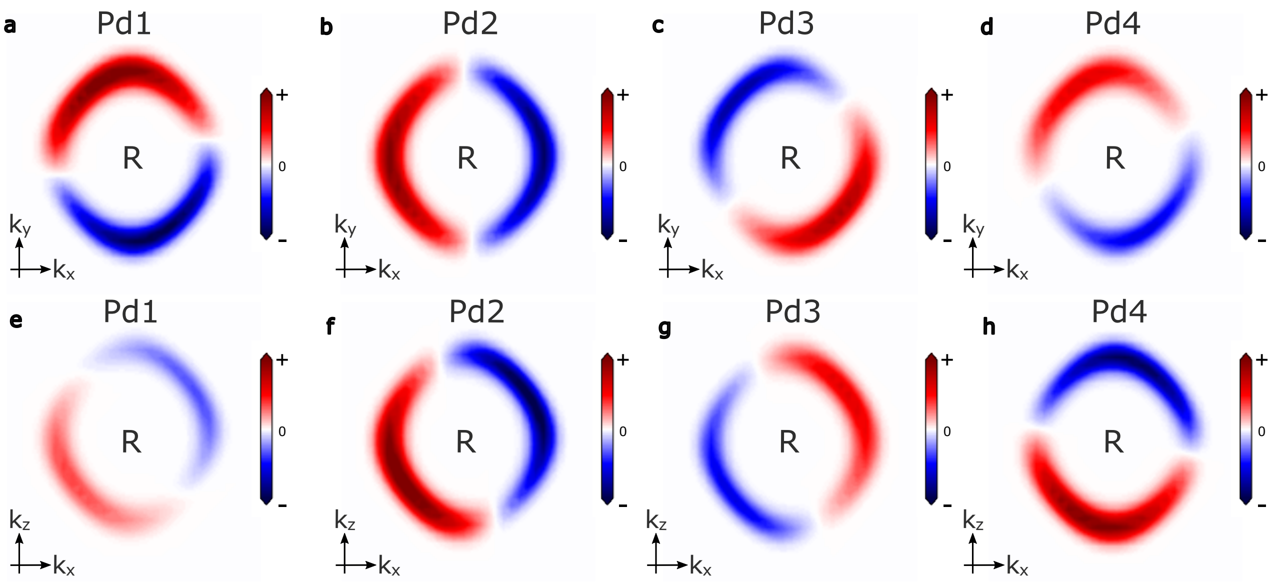

Supplementary Figure S5: **Local OAM projected onto incoming light direction for Pd1-Pd4** Local OAM projection for **a** – **d** $k_x - k_y$ plane and **e** – **h** $k_x - k_z$ plane are calculatd at 0.2 eV above the node. **a** and **c** are identical to the local OAM of Pd1 and Pd3 in Fig. 4**b** of the main text.

where the matrix element $M^+(\mathbf{k}, E)$ $[M^-(\mathbf{k}, E)]$ denotes the matrix element in the ACA with respect to the right-hand circularly (+) or left-and circularly (-) polarized light. The index $j = (a, m)$ includes atom site index (a) and atomic orbital index (m). The matrix $T_{jj'}(\mathbf{k}, E)$ includes all the matrix element and experimental geometry effects, with the expression

$$T_{jj'}(\mathbf{k}, E) = e^{-i\mathbf{k}\cdot(\mathbf{r}_j - \mathbf{r}_{j'})}[M_j^{(+)*}(\mathbf{k}, E)M_{j'}^{(+)}(\mathbf{k}, E) - M_j^{(-)*}(\mathbf{k}, E)M_{j'}^{(-)}(\mathbf{k}, E)] \quad \text{(Supplementary Equation S18)}$$

The full CD is then consist of intra-site local CD with the same atomic site $T_{amam'}$, and the inter-atomic interference terms with different atomic sites $T_{ama'm'}$ [CD simulation in Fig.4**b**–**c** in the main text].

The relation between local OAM and local intra-site CD depends on the details of the tensor $T_{jj'}(\mathbf{k}, E)$ and the atomic OAM matrix elements $L^{\mu}_{mm'}$. This also indicates that the correspondence between local OAM and CD is sensitive to the experimental geometry and the types of contributing orbitals. As an illustration, we calculate $T_{jj'}(\mathbf{k}, E)$ with photon energy $\omega = 400$ eV for d orbitals of a Pd atom, using the same experimental geometry as our PdGa(001) measurement. We can then relabel the CD tensor $T_{jj'}$ as $T_{mm'}$ with magnetic quantum numbers $m, m' = -2, \ldots, +2$. At Γ point, we find

$$T_{mm'} = \begin{bmatrix} -5.5 & 0.0 & 3.0i & -1.13 + 1.13i & 0.0 \\ 0.0 & 0.46 & -0.66 - 0.66i & 0.0 & -1.13 + 1.13i \\ -3.0i & -0.66 + 0.66i & 0 & -0.66 - 0.66i & -3.0i \\ -1.13 - 1.13i & 0 & -0.66 + 0.66i & -0.46 & 0.0 \\ 0 & -1.13 - 1.13i & 3.0i & 0.0 & 5.5 \end{bmatrix} \times 10^{-3} + O(10^{-5})$$

(Supplementary Equation S19)

If the quantization axis of the OAM operator is set to be along incoming light direction, then we have

$$L^{exp}_{mm'} = \begin{bmatrix} -2 & 0 & 0 & 0 & 0 \\ 0 & -1 & 0 & 0 & 0 \\ 0 & 0 & 0 & 0 & 0 \\ 0 & 0 & 0 & 1 & 0 \\ 0 & 0 & 0 & 0 & 2 \end{bmatrix} \quad \text{(Supplementary Equation S20)}$$

The proportionality between OAM and CD is then not guaranteed due to two reasons. First, $L^{exp}_{mm'}$ is diagonal, while $T_{mm'}$ has some non-zero off-diagonal terms. Second, $T_{m=m'=-2}(T_{m=m'=+2})$ has opposite sign to

$T_{m=m'=-1}(T_{m=m'=+1})$. Fortuanely, we find that at large photon energies (few hundred eV), the final state momentum is dominated by the component perpendicular to the surface, which leads to the dominating absolute value of the matrix elements $|T_{m=m'=\pm2}|$ compared to the other terms in $T_{mm'}$. This means that if the local OAM is mostly determined by $m = \pm2$ orbitals, the intra-site CD measurement is mostly proportional to the local OAM.

This is indeed the case for PdGa. In Fig.4**a** of the main text, we show the local OAM of both Pd1 and Pd3 are roughly proportional to the difference between weight of m= ±2 orbitals. As a result, the intra-site CD, which is calculated with the simplified phtoemission model, is roughly proportional to the local OAM in Fig.4**b**.

[S1] V. N. Strocov, X. Wang, M. Shi, M. Kobayashi, J. Krempasky, C. Hess, T. Schmitt, and L. Patthey, J. Synchrotron Radiat. **21**, 32 (2014).
[S2] H. Ebert, D. Ködderitzsch, and J. Minár, **74**, 096501 (2011).
[S3] J. Minar, J. Braun, S. Mankovsky, and H. Ebert, J. Electron Spectrosc. Relat. Phenom. **184**, 91 (2011).
[S4] G. H. Fecher, J. Kübler, and C. Felser, materials **15**, 5812 (2022).
[S5] J. P. Perdew, K. Burke, and M. Ernzerhof, Phys. Rev. Lett. **77**, 3865 (1996).
[S6] D. Xiao, M.-C. Chang, and Q. Niu, Rev. Mod. Phys. **82**, 1959 (2010).
[S7] J. Ma and D. A. Pesin, Phys. Rev. B **92**, 235205 (2015).
